# Disordered eating attitudes correlate with body dissatisfaction among Kuwaiti male college students

**DOI:** 10.1186/s40337-019-0265-z

**Published:** 2019-10-22

**Authors:** Mariam Ebrahim, Dalal Alkazemi, Tasleem A. Zafar, Stan Kubow

**Affiliations:** 1 Department of Food Science and Nutrition, College of Life Sciences, Kuwait University, P.O. Box 5969, 13060 Safat, Adailiya Kuwait; 2 School of Human Nutrition, McGill University, 21111 Lakeshore Road, Ste-Anne-de-Bellevue, Montreal, QC H9X3V9 Canada

**Keywords:** Feeding and eating disorders, Body weight, Body dysmorphic disorders, Body composition, Body mass index, Attitude

## Abstract

**Background:**

The prevalence of disordered eating attitudes and body dissatisfaction based on muscularity and body fat was investigated among male college students in Kuwait with a range of body mass index values including underweight, normal weight, overweight, and obese participants.

**Methods:**

Data were collected, using the Eating Attitudes Test (EAT-26) and the Bodybuilder Image Grid (BIG), from 400 male undergraduate students (84.8% Kuwaiti nationals) recruited from both public and private universities in Kuwait. An anonymous, self-administered questionnaire was used to determine the prevalence of symptomatology indicative of anorexia nervosa and bulimia nervosa and to examine the associations between body dissatisfaction and muscularity and body fat.

**Results:**

Most participants were dissatisfied with their current muscle mass and body fat (67.3 and 69%, respectively). Logistic regression analyses produced odds ratios (ORs) demonstrating that students dissatisfied with their muscularity and body fat and those who indicated a desire to decrease both muscularity and body fat had significantly higher odds of being at risk of disordered eating attitudes (OR = 2.241, 95% CI [1.17, 3.6], *p* = .032, and OR = 1.898, 95% CI [1.214, 2.967], *p* = .005, respectively). Obese participants also had higher odds of exhibiting disordered eating attitudes (OR = 2.06, 95% CI [1.17, 3.60], *p* = .011).

**Conclusion:**

The high proportion of disordered eating attitudes among Kuwaiti college men was associated with high levels of body image dissatisfaction in relation to both body fat and muscularity. High levels of eating disorder symptoms were also linked to obesity.

## Plain English summary

There is evidence of an increased risk of disordered eating attitudes among male college students in Kuwait; however, little is known as to how disordered eating attitudes are related to body dissatisfaction among Kuwaiti college males. The current study set out to examine male-focussed views of body dissatisfaction in terms of muscularity and body fat among Kuwaiti college males and how this is related to disordered eating attitudes including anorexia nervosa, bulimia nervosa, and binge-eating disorder. Although male college students demonstrated an objective view of their current weight status, more than 60 % of these males were noted to be dissatisfied with their level of both muscularity and body fat, with a greater degree of body dissatisfaction seen among obese students. An unrealistic desire among Kuwaiti college men to increase muscle mass as well as drastically decrease body fat was related to the risk of disordered eating attitudes, which is a situation that among Kuwaiti college men and may be further can worsen without professional intervention. Greater education is required in Kuwaiti campuses to promote healthy body image awareness and to adopt healthy weight-related behaviors among Kuwaiti college males.

## Background

Body image dissatisfaction and the prevalence of extreme body shape and weight control behaviors are reported to be increasing among college students of both genders [40]. College-aged female students often show a strong desire for thinness and use dieting and food control behaviors to lose weight and achieve their desired body image [5]. Measures for assessment of eating disorders in men, however, have been relatively under-investigated [36]. Eating disorder assessments can exhibit gender bias as men and women differ in their primary body concerns. Male-specific behaviors can arise from body image disturbances related to a drive for increased “leanness” focussed upon muscularity and reduced body fat [56]. Male college students and athletes tend to strive for a more muscular physique over thinness, which is often based on distortions in perceived ideal body image of fat and muscle levels [32].

The muscular physique has attracted more interest among males as their participation in bodybuilding has increased; this practice can lead to experiencing negative thoughts relating to body image [43]. Body image dissatisfaction is increasingly observed among men and adolescent boys worldwide affecting their quality of life [17, 30, 40, 60, 62]. Body image dissatisfaction negatively influences self-esteem causing long-lasting depression [12] and muscle dysmorphia [29, 38]. Muscle dysmorphia could compel men to use steroids leading to substance abuse and increased suicide attempts [11]. Body image dissatisfaction among college students is associated with an increased risk of disordered eating attitudes and eating disorders [39]. An increasing prevalence of male body dissatisfaction and extreme body shape and weight control behaviors have been reported [40].

The relatively high obesity prevalence in Kuwait is mirrored by an alarmingly high prevalence of disordered eating attitudes among young people, which exceeds the prevalence reported worldwide and in neighbouring Arab countries [45]. Musaiger et al. [47] reported that the prevalence of disordered eating attitudes in Kuwaiti adolescents is 47% among boys and 43% among girls, whereas the prevalence ranges from 14.6 to 23.2% in other Arab countries, including Syria, Jordan, and Libya. The higher prevalence of disordered eating attitudes among Kuwaiti adolescents may be due to its culture, which is generally considered much more open than many other Arab countries, with a relatively liberal society and extravagant wealth [45]. The rapid socio-economic changes in Kuwait since early 1990’s shifted the attitudes and behaviors of adolescents to more Western values, such as developing keen interest in global fashion trends and perceiving thinness (for girls) and (leanness for boys) as ideal body shape [48]. There is an increased pressure from mass media, more specifically internet exposure, on body weight concerns that can distort adolescent body perceptions and may lead them to practice unhealthy weight control behaviors [54].

Among 530 college students recruited from Kuwait University and two other private colleges, Musaiger et al. [45] found the prevalence of disordered eating attitudes to be 33.6% in male students and 31.8% in female students, which is higher than observed in college students from universities in Western countries. For example, Eisenberg, Nicklett, Roeder, & Kirz [21] found that 13% of female and 3.6% of male US college students have disordered eating attitudes, while Jacobi et al. [33] found that 11.2% of female college students in Germany were at risk of disordered eating attitudes.

Although a higher risk of disordered eating attitudes is associated with obesity, some studies report an inconsistent relationship between disordered eating attitudes and weight status. A recent study of Kuwaiti female students using the Eating Attitudes Test (EAT-26) revealed that all weight categories exhibited disordered eating attitudes and distortions in weight perception [5]. There are many other potential risk factors associated with disordered eating attitudes and behaviors among young people including nutrition and cultural transition, social changes, Westernization, family environment, parenting style, exposure to mass media, and globalization.

To date, the risk of disordered eating attitudes among male Kuwaiti college students has not been studied including the association of this risk with muscularity and body fat. A primary goal of the present study was to assess body image disturbances in Kuwaiti male college-aged students using the Bodybuilder Image Grid (BIG). The EAT-26 test screened male Kuwaiti college students for their risk of developing an eating disorder. A secondary objective was to determine what male college students in Kuwait consider to be the ideal male body type with regards to body fat and muscularity. Accordingly, we hypothesized that men who reported more body dissatisfaction according to BIG, i.e., those striving for a more muscular body ideal as opposed to those with current thinner or fatter body type, would report greater disordered eating attitudes as measured by the EAT-26.

## Methods

This cross-sectional descriptive study included 400 male undergraduate students as participants, recruited from public and private universities in Kuwait, including Kuwait University (five campuses, 15 colleges), the Gulf University for Science and Technology, and the Australian College of Kuwait. Data were collected between September 2015 and February 2016 using self-administered anonymous questionnaires. Students were invited to participate in the study voluntarily during breaks between classes. During recruitment, the purpose and format of the questionnaire were explained verbally to participants. All relevant information about participation was provided clearly in writing as a preface to each questionnaire. Before the start of the study, ethical approval was obtained from the Kuwait University Research Sector and Campuses Administration Office, which provided access to all campuses for participant recruitment. Ethical approval was also obtained from the Directors of Students’ Affairs at the Gulf University for Science and Technology and at the Australian College of Kuwait. Consent to publish and report individual data was obtained from all participants. Students under 18 years of age and participants who provided incomplete questionnaires were excluded.

### Main outcome variables

#### Disordered eating attitudes

Disordered eating attitudes were measured using the EAT-26, which has high sensitivity and reliability (a Cronbach’s alpha of .80) both in participants with eating disorders and in those with normal eating behaviour [27]. The EAT-26 comprises 26 items on three subscales: dieting (Sub-D; contains 13 items relating to distortion of body image); bulimia (Sub-B; contains six items on body image and tendency toward bulimic behaviour); and oral control (Sub-O; contains seven items on self-control and high-risk behaviors associated with anorexia nervosa). Each item of the EAT-26 is rated on a four-point Likert scale (0 = *sometimes, rarely, or never*; 1 = *often*; 2 = *usually*; 3 = *always*). Total scores on the EAT-26 range from 0 to 78. An EAT-26 score ≥ 20 indicates possible anorexia nervosa or bulimia nervosa while a score ≥ 11 indicates possible binge-eating disorder. There are no cut-off points relating specifically to any of the EAT-26 subscales, although Garfinkel and Newman [26] propose that scores > 20 on Sub-D and > 10 on the other two subscales indicate a high risk of developing an eating disorder. Each participant completed either an Arabic or an English version of EAT-26. The Arabic version of EAT-26 was validated by Al-Subaie et al. [9]. Its sensitivity and specificity were 100 and 84.6%, respectively. EAT-26 was applied among adolescents aged between 12 and 18 years in some Arab countries including Egypt, Jordan, Lebanon, Saudi Arabia, Oman, and the United Arab Emirates [10, 20, 23, 46, 50, 61]. Recently, this version has been also used among undergraduate students in Kuwait [45], although it has not been validated in Kuwait.

#### Body fat and muscularity dissatisfaction

The Bodybuilder Image Grid (BIG), which assesses body dissatisfaction-related indices pertaining to muscularity, thinness, and fatness [32]. BIG allows individuals to select male silhouettes with varying degrees of both muscle mass and body fat level, which are essential components of body image disturbances in men. Figure-rating scales such as BIG are highly correlated with self-reported body mass index (BMI [37];), and can detect body-related attitudinal and perceptual disturbances including body dissatisfaction and body image distortions [52]. This measure consists of 30 male silhouettes with varying levels of muscle and body fat, displayed in a grid. The figure at the top left of the grid represents the man with the least body fat and the least muscle mass. From left to right across the columns, the figures increase in body fat levels, starting from a value representing extremely low body fat and reaching a value representing extremely high body fat. Each column represents a 6.5% stepwise increase in body fat with figures in column 1 illustrating 3.5% body fat, followed by 10, 16.5, 23, 29.5, and 36% in columns 2 to 6, respectively. From top to bottom going down the rows of the grid, the figures increase in muscle mass, or fat-free mass index (FFMI). The figures in row 1 represent extremely low muscle mass (FFMI 15.5) while those in rows 2, 3, and 4 represent muscle mass of 18.9, 22, and 25.6 FFMI, respectively. The figures in row 5 represent extremely high muscle mass (FFMI 29). The FFMI was calculated as follows: W × [(100 – (% body fat)) / 100] × *H*^2^ + [6.1 × (1.8 – H)]. “W” is weight in kilograms, and “H” is height in meters. A FFMI greater than 25 represents a level of muscle mass that is only achievable with the use of anabolic-androgenic steroids (AAS). Participants indicated their current body type and their ideal body type by selecting the figures in the grid. Body fat dissatisfaction was computed by subtracting the body fat level of the participant’s current body type selection from the body fat level of their ideal body type selection (body fat dissatisfaction = current fat – ideal fat). Higher positive numbers indicated a stronger desire to be leaner. Muscularity dissatisfaction was calculated by subtracting the participant’s indicated current muscle mass from their indicated ideal muscle mass. A score of zero indicated that the participant was satisfied with his current muscle mass, a negative score indicated a desire to increase muscle mass, and a positive score showed a want to decrease muscle mass. BIG has shown excellent test-retest reliability, internal consistency, and convergent and divergent validity [32].

### Weight status

Participants were grouped according to the following self-reported BMI (kg/m^2^) categories: < 18.5 = underweight, 18.5–24.9 = normal weight, 25.0–29.9 = overweight, ≥ 30.0 = obesity.

### Covariates

The following variables were included as potential covariates of disordered eating attitudes as determined by previous studies in Kuwait [5, 45]: nationality (Kuwaiti vs. non-Kuwaiti), age (continuous variable), marital status (single vs. married), field of study (science-related major vs. arts-related major), and university type (public vs. private).

### Statistical analysis

Anthropometric data and eating behavior scores were checked for normality before being treated as continuous variables in the analysis. EAT-26 scores and subscale scores were compared between participants falling into the following two sets of binary categorical variables using Student’s *t*-tests: (a) at risk of disordered eating vs. not at risk of disordered eating to confirm that the two groups were statistically different from each other; and (b) overweight or obese vs. underweight or normal weight. Comparisons among multinomial variables, including the four BMI categories and body dissatisfaction groups, according to body size, muscularity, and body fat, were performed using analyses of variance. Cross-tabulations (chi-squared tests) were performed to identify differences in the prevalence of risk of disordered eating (based on EAT-26 and subscale scores) according to BMI category (weight status), body dissatisfaction, and covariates. All *p* values reported are two-tailed, and an alpha level of .05 was considered to be the threshold for significance for all tests performed.

Unadjusted logistic regression analyses were conducted to generate prevalence odds ratios (ORs) with 95% confidence intervals (CIs) for the following outcomes: (a) risk of disordered eating attitudes (EAT-26 score ≥ 20); (b) risk of extreme dieting behaviors (Sub-D score ≥ 20); (c) risk of bulimic tendencies (Sub-B score ≥ 10); and (d) restrictive oral control behaviors associated with anorexia nervosa (Sub-O score ≥ 10). Body fat and muscularity dissatisfaction were included as independent variables. All statistical analyses were performed using SPSS Statistics 23.0 (IBM Corp., Armonk, NY).

## Results

### Participant characteristics

Participant characteristics are shown in Table 1. A total of 498 male students were approached, of whom 419 agreed to participate. A total of 400 participants (95.46%) completed EAT-26 and provided self-assessed body weight and height measurements. Both types of academic majors were represented equally among the participants (i.e., 50% science-related majors and 50% arts-related majors). Students from two private colleges and all Kuwait University colleges were represented. The majority of participants were Kuwaiti (84.4%). With regards to non-Kuwaiti students, they comprised 15.5% (*n* = 62) of the total population, with 2 students from USA, 1 Austrian, 2 Bahraini, 3 Canadian, 10 Egyptians, 1 Iranian, 1 Iraqi, 15 Jordanians, 6 Lebanese, 1 Lithuanian, 2 Palestinians, 1 Russian, 8 Saudis, 1 Sudanese, 7 Syrians, and 1 Tunisian.

**Table 1:** Anthropometrics and EAT-26 scores (*N* = 400)

Independent variables	Total *n* (%) or *M* (SD)	EAT-26 categories		
		At risk of disordered eating attitudes (*n* = 185, 46.2%)	Not at risk of disordered eating attitudes (*n* = 215, 53.8%)	*t(d.f.)*, *p*, *d* or *χ*^2^ test
Age (years)	21.9 (3.2)	22.1 (3.18)	21.76 (3.2)	1.07 (398), .28, .106
Self-reported weight (kg)	79 (16.9)	81.75 (17.6)	76.64 (15.9)	3.04 (398), .002, .304
Self-reported height (cm)	174.8 (7)	175.17 (6.99)	174.56 (7.0)	0.85 (398), .391, .087
Body mass index (BMI)	25.8 (5.3)	26.6 (5.7)	25.1 (4.9)	2.9 (398), .004, .174
BMI category (*n*)				
Underweight (BMI < 18.5)	19 (4.8%)	6 (3.2%)	13 (6%)	χ^2^(3) = 9.39, *p* < .024
Normal weight (18.5 ≤ BMI ≤ 24.9)	178 (44.5%)	71 (38.4%)	107 (49.8%)	
Overweight (25 ≤ BMI ≤ 29.9)	132 (33%)	67 (36.2%)	65 (30.2%)	
Obese (BMI ≥ 30)	71 (17.8%)	41 (22.2%)	30 (14%)	
Eating attitudes				
EAT-26 (total score)	20.4 (14.1)	32.7 (10.8)	9.87 (5.1)	27.58 (398), .004, .80
EAT-26 Dieting (score)	10.99 (8.0)	17.8 (6.0)	5.1 (3.8)	25.5 (398), < .001, .78
EAT-26 Bulimia (score)	3.85 (3.9)	6.6 (3.96)	1.48 (1.8)	17.1 (398), < .001, .56
EAT-26 Oral control (score)	5.6 (4.28)	8.3 (4.12)	3.27 (2.8)	14.5 (398), < .001, .58
Nationality (*n)*				
Kuwaiti	339 (84.8%)	162 (87.6%)	177 (82.3%)	χ^2^(1) = 2.11, *p* < .146
Non-Kuwaiti	61 (15.3%)	23 (12.4%)	38 (17.7%)	
University type (*n*)				
Public	200 (50%)	85 (45.9%)	115 (53.5%)	χ^2^(1) = 2.26, *p* < .160
Private	200 (50%)	100 (54.1%)	100 (56.5%)	
Major field of study (n)				
Science-related	200 (50%)	77 (41.6%)	123 (57.2%)	χ^2^(1) = 9.66, *p* < .003
Arts-related	200 (50%)	108 (58.4%)	92 (42.8%)	
Muscle mass satisfaction (n)				
Satisfied	131 (32.8%)	58 (31.4%)	73 (34%)	χ^2^(2) = 5.54, *p* < .063
Desire to increase	230 (57.5%)	102 (55.1%)	128 (59.5%)	
Desire to decrease	39 (9.8%)	25 (13.5%)	14 (6.5%)	
Body fat satisfaction (n)				
Satisfied	124 (31%)	46 (24.9%)	78 (36.3%)	χ^2^(2) = 9.48, *p* < .009
Desire to increase	45 (11.2%)	17 (9.2%)	28 (13%)	
Desire to decrease	231 (57.8%)	122 (65.9%)	109 (50.2%)	

EAT-26 = Eating Attitudes Test. Pearson’s chi-squared test was used to compare the prevalence of the risk of disordered eating between groups in relation to BMI category, nationality, type of university, and major field of study

Half of the students were categorized as overweight or obese (33 and 17.8%, respectively) while 4.8% were underweight and 44.5% were normal weight (Table 1). The mean BMI was 25.83 (*SD* = 5.31), which falls within the overweight category (Table 1).

### Disordered eating attitudes and subscales

Among all participants (*n* = 400), the mean total EAT-26 score was 20.4 (*SD* = 14.08; Table 1). The proportion of participants identified to be at risk of disordered eating attitudes (i.e., with an EAT-26 score equal to or above the diagnostic cut-off of 20; Table 1) was 46.2% (*n* = 185). The mean EAT-26 score was significantly higher among the group at risk of disordered eating attitudes than among the not-at-risk group (*M* = 32.7, *SD* = 10.8 vs. *M* = 9.87, *SD* = 5.13; *t* (398) = 27.58, *p* = .004, *d* = .80). The mean score for each subscale was higher in the at-risk group than in the not-at-risk group; however, neither of the group mean subscale scores reached the proposed cut-off value for increased risk (Table 1). In terms of the proportion of participants with scores greater or equal to the proposed cut-off values for each of the EAT-26 subscales, 16.8% (*n* = 67) scored ≥20 on the Sub-D scale (dieting), 11.2% (*n* = 45) scored ≥10 on the Sub-B scale (bulimia), and 19% (*n* = 76) scored ≥10 on the Sub-O scale (oral control).

### Covariates and disordered eating attitudes

The proportion of students at risk of disordered eating attitudes was higher among those at the private universities (54.1%) than at the public university (45.9%); however, the difference was not statistically significant (*p* = .16; Table 1). The proportion of students at risk of disordered eating attitudes was higher among those with arts-related majors than the science-related majors (58.4% vs. 41.6%, χ^2^(1) = 9.66, *p* <.001; Table 1). The proportion of participants at risk of disordered eating attitudes did not differ according to nationality or marital status. The mean BMI of the group at risk of disordered eating attitudes did not differ from that of the not-at-risk group, with the mean BMI for both groups falling within the overweight category (Table 1). A higher proportion of participants were at risk of disordered eating attitudes among those who were overweight than among those who were obese (36.2% vs. 30.2, and 22.2% vs. 14%, χ^2^(3) = 9.39, *p* = .024).

### Muscle mass dissatisfaction and disordered eating attitudes

**Fig. 1:**
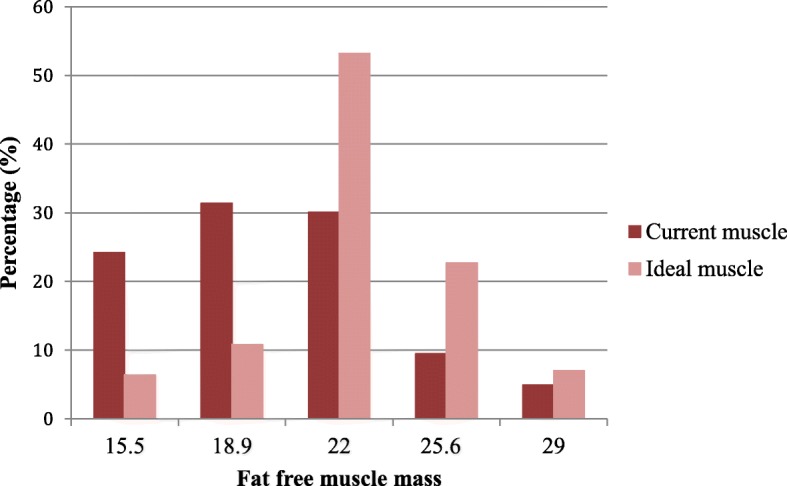
Histogram indicating the frequency with which participants selected each fat free muscle mass category in the Bodybuilder Image Grid to represent their current and ideal muscle mass

The majority of participants (67.3%) were dissatisfied with their current muscle mass as more than half of all participants (57.5%) wished to increase their muscularity, with no difference for such a desire between the group with at-risk of disordered eating attitudes and the not-at-risk group (55.1% vs. 59.5%, *p* = .063). A smaller proportion of participants (9%) wished to decrease their muscle mass with a higher proportion of participants with such a desire in the at-risk group (13.5% vs. 6.5%, χ^2^(2) = 5.54, *p* = .063). Only 32.8% of participants were satisfied with their current muscle mass. Most participants (87.7%) indicated that their current muscle mass was 22 or less, and a similarly high percentage (82.4%) indicated an ideal muscle mass ≥ 22 (χ^2^(4) = 12.42, *p* = .014; Table 2 and Fig. 1). Almost one-third of participants (29.8%) indicated an ideal body with an FFMI > 25. Participants with a current muscle mass > 22 were found to be more likely to be at risk of disordered eating attitudes (FFMI 25.6: 60.6% of participants; FFMI 29: 75% of participants) compared to those with a lower current muscle mass (Table 2). Similarly, a higher proportion of those with a desire for a muscle mass > 22 were found to be at risk of disordered eating attitudes (FFMI 25.6 = 52.7% and FFMI 29 = 65.4%; Table 2 and Fig. 1). The mean BMI of participants at each muscle mass increased gradually from 25 at 15.5% to 29.56 at 29% (Table 2). In contrast, the mean BMI did not vary across the range of ideal muscle mass categories, which indicated that there was no relationship between BMI and ideal muscle mass.

**Table 2:** Participants’ current and ideal muscle mass with prevalence of disordered eating attitudes and body mass index for each category

Muscle mass (FFMI)	Total selections *n* (%)	BMI *M* (*SD*)	EAT-26 categories		
			At risk of disordered eating attitudes *n* (%)	Not at risk of disordered eating attitudes *n* (%)	χ^2^(d.f.), *p*
Current					
15.5	99 (24.8%)	25 (5.77)	38 (38.4%)	61 (61.6%)	χ^2^(4) = 12.42, *p* < .014
18.9	128 (32.1%)	25.53 (4.7)	53 (41.4%)	75 (58.6%)	
22	123 (30.8%)	25.97 (4.54)	62 (50%)	62 (50%)	
25.6	33 (8.3%)	26.93 (6.17)	20 (60.6%)	13 (39.4%)	
29	16 (4%)	29.56 (8.67)	12 (75%)	4 (25%)	
Ideal					
15.5	27 (6.8%)	25.14 (5.85)	12 (44.4%)	15 (55.6%)	χ^2^(4) = 7.24, *p* < .124
18.9	43 (10.8%)	24.01 (5.55)	19 (44.2%)	24 (55.8%)	
22	210 (52.6%)	26.11 (5.27)	88 (41.7%)	123 (58.3%)	
25.6	93 (23.3%)	26.11 (5.36)	49 (52.7%)	44 (47.3%)	
29	26 (6.5%)	26.24 (4.09)	17 (65.4%)	9 (34.6%)	

*BMI* body mass index, *EAT-26* eating attitudes test, *FFMI* fat free mass index. Pearson’s chi-squared tests were used to compare the prevalence of the risk of disordered eating between groups in relation to current and ideal levels of muscle mass

### Body fat dissatisfaction and disordered eating attitudes

**Fig. 2:**
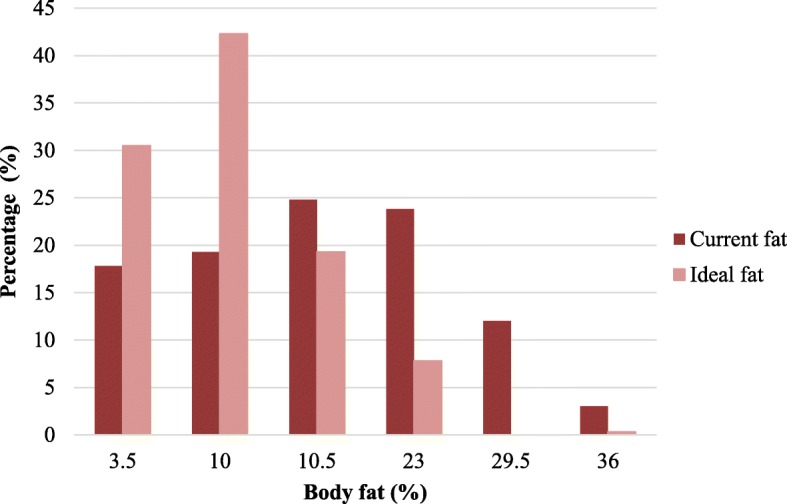
Histogram indicating the frequency with which participants selected each body fat category on the Bodybuilder Image Grid to represent their current and ideal fat levels

The majority of participants (69%) were dissatisfied with their current level of body fat with more than half (57.8%) wishing to decrease their body fat. Among those with such a desire, a larger proportion of participants were found in the at-risk of disordered eating attitudes group compared to those without such a desire (65.9% vs. 50%, χ^2^(2) = 9.48, *p* = .009). One-third of participants (31%) were satisfied with their current level of body fat, and 11.2% wished to increase their body fat (Table 1). Almost half of the participants (48.6%) indicated that a silhouette with either 16.5% body fat (24.8% of participants) or 23% body fat (23.8% of participants) best reflected their current body fat whereas most participants (72.7%) indicated an ideal body fat level ≤ 10% (χ^2^(4) = 4.56, *p* = .335; Table 3 and Fig. 2).

**Table 3:** Participants’ current and ideal body fat with prevalence of disordered eating attitudes and body mass index for each category

Body fat level (%)	Total selections *n* (%)	BMI *M* (*SD*)	EAT-26 categories		
			At risk of disordered eating attitudes *n* (%)	Not at risk of disordered eating attitudes *n* (%)	χ^2^(d.f.), *p*
Current					
3.5	71 (17.8%)	22.73 (3.66)	23 (31.9%)	49 (68.1%)	χ^2^(5) = 10.20, *p* < .070
10	77 (19.3%)	22.81 (3.44)	33 (42.9%)	44 (57.1%)	
16.5	99 (24.8%)	24.78 (3.5)	48 (48.5%)	51 (51.5%)	
23	95 (23.8%)	27.89 (3.8)	50 (52.6%)	45 (47.4%)	
29.5	48 (12%)	31.65 (5.58)	25 (52.1%)	23 (47.9%)	
36	9 (2.3%)	34.88 (11.58)	6 (66.7%)	3 (33.3%)	
Ideal					
3.5	122 (30.5%)	25.04 (5.00)	62 (50.8%)	60 (49.2%)	χ^2^(4) = 4.56, *p* < .335
10	169 (42.2%)	25.73 (5.19)	73 (43.2%)	96 (56.8%)	
16.5	77 (19.2%)	26.96 (5.63)	39 (50.6%)	38 (49.4%)	
23	31 (7.8%)	26.63 (6.01)	11 (35.5%)	20 (64.5%)	
29.5	0 (0.0%)	–	0 (0.0%)	0 (0.0%)	
36	1 (0.2%)	26.7	0 (0.0%)	1 (100%)	

*BMI* body mass index, *EAT-26* eating attitudes test. Pearson’s chi-squared test was used to compare the prevalence of the risk of disordered eating between groups in relation to current and ideal levels of body fat

### Associations between disordered eating attitudes and independent variables

A high prevalence of disordered eating attitudes was observed for all BMI groups, including among participants with a normal weight (BMI 18.5–24.9). More than one-third (39.9%) of all participants had a mean of EAT-26 score equal to or exceeding the cut-off value of 20; the proportions of participants in the underweight, overweight, and obese categories scoring above the cut-off were 31.6, 50.8, and 57.7%, respectively. Participants who were obese (BMI ≥ 30) had higher odds of being at risk of disordered eating attitudes (OR = 2.06, 95% CI [1.17, 3.60], *p* = .011). Participants with a BMI ≥ 30 had higher odds of engaging in dieting behaviors (OR = 2.063, 95% CI [1.01, 4.21], *p* = .043) compared to those at a normal weight. Being in either the underweight or the obese BMI group increased odds of restrictive oral control behaviors associated with anorexia nervosa (OR = 2.56, 95% CI [1.08, 6.04], *p* = .018, and OR = 8.22, 95% CI [2.49, 27.0], *p* = .031, respectively; Table 4). Being dissatisfied with one’s muscle mass or body fat and wishing to reduce them was associated with significantly higher odds of disordered eating attitudes (OR = 2.241, 95% CI [1.17, 3.6], *p* = .032, and OR = 1.898, 95% CI [1.214, 2.967], *p* = .005, respectively). The desire to decrease muscle mass was associated with significantly higher odds of a tendency to bulimic behaviors (OR = 2.7, 95% CI [1.064, 6.969], *p* = .037). Participants who wished to decrease their body fat were less likely to engage in oral control (OR = 0.531, 95% CI [0.302, 0.936], *p* = .029; Table 4).

**Table 4:** Prevalence of and odds ratios for risk of disordered eating attitudes according to EAT-26 and Subscale Scores (*N* = 400)

Risk factor	Category	%	OR [95% CI]	*p*
Dependent variable: at risk of disordered eating attitudes (EAT-26 ≥ 20)				
BMI category	BMI 18.5–24.9	39.9	1	
	BMI < 18.5	31.6	0.69 [0.25, 1.91]	.482
	BMI 25–29.9	50.8	1.55 [0.98, 2.44]	.057
	BMI ≥ 30	57.7	2.06 [1.17, 3.60]	.011
Muscle mass satisfaction	Satisfied	44.3	1	
	Desire to increase	44.3	1 [0.651, 1.54]	.989
	Desire to decrease	64.1	2.241 [1.073, 4.7]	.032
Body fat satisfaction	Satisfied	37.1	1	
	Desire to increase	37.8	1.030 [0.509, 2.082]	.936
	Desire to decrease	52.8	1.898 [1.214, 2.967]	.005
Dependent variable: EAT-26 Dieting subscale				
BMI category	BMI 18.5–24.9	12.4	1	
	BMI < 18.5	10.5	0.834 [0.18, 3.85]	.817
	BMI 25–29.9	20.5	1.823 [0.99, 3.37]	.056
	BMI ≥ 30	22.5	2.063 [1.01, 4.2]	.047
Muscle mass satisfaction	Satisfied	19.8	1	
	Desire to increase	13.5	1.59 [0.355, 1.115]	.112
	Desire to decrease	25.6	1.393 [0.603, 3.217]	.438
Body fat satisfaction	Satisfied	16.9	1	
	Desire to increase	20	1.226 [0.515, 2.92]	.645
	Desire to decrease	16	.935 [0.520, 1.681]	.823
Dependent variable: EAT-26 Bulimia subscale				
BMI category	BMI 18.5–24.9	9.6	1	
	BMI < 18.5	5.3	0.52 [0.06, 4.19]	.544
	BMI 25–29.9	12.1	1.30 [0.63, 2.69]	.469
	BMI ≥ 30	15.5	1.73 [0.76, 3.92]	.184
Muscle mass satisfaction	Satisfied	9.9	1	
	Desire to increase	10	1 [0.493, 2.065]	.981
	Desire to decrease	23.1	2.7 [1.064, 6.969]	.037
Body fat satisfaction	Satisfied	14.5	1	
	Desire to increase	11.1	0.736 [0.256, 2.11]	.569
	Desire to decrease	9.5	0.620 [0.319, 1.206]	.159
Dependent variable: EAT-26 Oral control subscale (*anorexic tendencies*)				
BMI category	BMI 18.5–24.9	21.9	1	
	BMI < 18.5	47.4	2.56 [1.08, 6.04]	.018
	BMI 25–29.9	15.9	8.22 [2.49, 27.0]	.188
	BMI ≥ 30	9.9	1.73 [0.69, 4.29]	.031
Muscle mass satisfaction	Satisfied	16.8	1	
	Desire to increase	18.7	1.139 [0.647, 2]	.651
	Desire to decrease	28.2	1.946 [0.845, 4.484]	.118
Body fat satisfaction	Satisfied	22.6	1	
	Desire to increase	37.8	2.082 [0.998, 4.342]	.051
	Desire to decrease	13.4	0.531 [0.302, 0.936]	.029

*EAT-26* eating attitudes test, *BMI* body mass index, *OR* odds ratio, *CI* confidence interval. The third column indicates the prevalence of risk, as indicated by each dependent variable, within each category of the relevant risk factor. Odds ratios with *p* < .05 are taken to indicate significant differences in risk

## Discussion

This is the first large-scale study to quantify disordered eating attitudes and its subscales and body dissatisfaction based on muscularity and body fat among Kuwaiti college-aged men. The high prevalence of disordered eating attitudes among male college students in Kuwait (46.2%) is consistent with previous observations of Kuwaiti college-aged women (46.4% [5];) and Kuwaiti adolescent boys (47%) [47]. The prevalence of disordered eating attitudes is the highest reported to date among males in Gulf Cooperation Council [57] and other Arab countries, which might be due to the relatively high prevalence of overweight and obesity in Kuwait [3]. In Kuwait, the proportions of overweight and obesity are considered among the highest in the world. Using BMI, 78% of men and 82% of women in Kuwait were overweight or obese [51]. The overall obesity prevalence in Kuwait was reported to be at 42% compared to 36% in Saudi Arabia, 34% United Arab Emirates, and 26% in Qatar [6]. According to WHO Global Health Observatory for 2016 [64], 37.9% of Kuwaiti adults are obese, while the prevalence of obesity is 35.5% in Saudi Arabia, 31.7% in the United Arab Emirates, and 35.1% in Qatar. In comparison, the prevalence of obesity was noted to be 36.2% in the USA, 27.8% in UK, 29.4% in Canada, and 29% in Australia.

The prevalence of disordered eating attitudes was also much higher than prevalence reported among college-aged men in Spain (14.9% [58];) and Malaysia (13.5% [25];). The current study, however, showed a higher prevalence of disordered eating attitudes than reported previously in 2010 among male Kuwaiti college students (31.8%) [45]. This disparity may be attributed to differences in the sample size and sampling methods, which affect prevalence estimates. The Musaiger et al. [45] study used a convenience sample of 203 male participants recruited from two public colleges and two private universities whereas the current study employed proportional sampling methods to obtain a more representative sample of Kuwaiti college men from all the public colleges of Kuwait University (15 colleges) and two private universities. The disordered eating attitudes in Kuwaiti college-aged men shown in the current work is comparable to previous observations of Kuwaiti college-aged women [5] as 16.8% of men reporting extreme dieting behaviors to control their weight compared to 15.9% of women, 11.2% exhibited bulimic tendencies versus 9.9% among women, and 19% showed behavioral tendencies toward anorexia nervosa compared to 18.3% among women. Taken together, these findings suggest that disordered eating attitudes are common among college students in Kuwait with a similar burden in both genders.

The motivation behind these behaviors, however, may differ between genders. As described by Ralph-Neaman & Filik [53], the motivation to lose fat has two dimensions, the drive for thinness and the drive for leanness. The drive for thinness is reported as more focused on the fear of gaining any weight and determination to be thin, whereas the drive for leanness is focused on attitudes which are not directly pertaining to fat, but a desire to lose fat and gain a toned, muscular, and lean body. Men tend to strive toward leanness (i.e., a lean and toned body) rather than either a larger, muscular body or the thin ideal that many women tend to strive toward, i.e., “the drive for thinness” [53]. In the present study, the BIG data showed that 67.3% of men were dissatisfied with their current muscle mass with 57.5% wishing to increase their muscle mass and 9.8% desiring to decrease their muscle mass. Most men (69%) were dissatisfied with their current level of body fat as 57.8% wished to decrease their body fat whereas 11.3% had a desire to increase their body fat. Accordingly, it appears that Kuwaiti college-aged men generally desired an ideal body type that was significantly more muscular and leaner than their current body type. The majority of the participants selected their current body fat level between 3.5 and 23% body fat with an ideal fat level ≤ 16.5%, with the most common preference for 10% body fat. Most men indicated that their current muscle mass was between FFMI of 15.5 and 22 with 52.6% selected 22 as their ideal muscle mass, which is higher than the FFMI of 20 reported for the average American man. A concern was those who desired a muscle mass of FFMI of 25.6 or more, which is a level that can only be achieved with the aid of AAS [32]. Muscle dissatisfaction in men has been linked to poorer self-esteem and higher levels of life dissatisfaction and depression [13]. As reviewed by Devrim, Bilgic, and Hongu [19], male body image disturbance can lead to abuse of drugs [18], social isolation due to prioritizing gym time over time with family and friends [22], hiding in baggy clothes, career deterioration, depression, and suicide [11].

There are many differences between Kuwaiti students and the American or students from the Western countries including religion, urbanization, socio- political factors, family structure, and family size. Recently, Gorrell, Trainor, and Le Grange [16] identified the impact of urbanization on eating disorders and highlighted specific risk factors for eating disorders, including acculturation to Western standards of beauty and food resources with associated body weight and body image concern. Furthermore, the cultural framework of the American population is individualism, where the cultural framework is collectivism for many Asian, African, and Hispanic nations, including Kuwait [63]. Individualism is positively correlated with high self-esteem [31], and self–esteem is strongly and positively correlated with mental health [1]. Abdel-Khalek & Lester [2] observed that Kuwaiti college students obtained slightly lower mean score on the Mental Health Scale, compared to American. This difference was attributed to the different levels of freedom, individualism, and collectivism between the two groups of students. In comparing Kuwaiti and American college students, previous results suggested significantly higher mean scores for Kuwaiti college students than did their American counterparts on measures of anxiety, depression, obsession-compulsion, pessimism, ego-grasping, and death obsession. On the other hand, American college students obtained significantly higher mean scores than did their Kuwaiti peers on optimism, love of life, and happiness. Collectively, these factors make Kuwaiti students more likely have cultural adjustment difficulties, which may increase their distress and lead to problems such as disordered eating attitudes.

In the present study, generally men who fell into the obese BMI category wished to reduce both their body fat and their muscle mass. Such participants were also found to have higher odds of exhibiting restrictive dieting behaviors and tendencies to bulimia. Our data showed that college-aged men who wished to decrease their body fat were found to have lower odds of anorexic tendencies; however, college-aged men who fell into the underweight category had ~ 2.5 times higher odds of anorexic tendencies compared to those at a normal weight. This pattern is similar to our previous findings that underweight college-aged women had more than twice the odds of exhibiting restrictive oral control behaviors associated with anorexia nervosa compared to those who are not underweight [5]. These findings show that those who are underweight may use extreme oral restrictive methods to achieve their desired weight, whereas heavier students may not be interested in dietary restrictions and use other means to purge excess calories such as in the bulimic subtype. Al-Sejary [8] showed similar reluctance towards dietary restrictions among Kuwaiti students with higher weight status and with the metabolic syndrome. More studies are needed to elucidate further on the motivators of behaviors and dietary restrictions.

Overall, the present findings are in line with previous work suggesting an increasingly high prevalence of male body dissatisfaction and extreme body shape and weight control behaviors [40]. Murray et al. [44] reported that male participants who are dissatisfied with their current body shape as assessed by BIG are more likely to exhibit disordered eating attitudes and behaviors towards changing their body shape to achieve their desired body image. Devrim et al. [19] showed that 53.7% of Turkish male bodybuilders exhibited body dissatisfaction using the BIG survey and that body dissatisfaction was significantly correlated with eating disorder tendencies according to EAT-40 scores. In a study using the Figure Rating Scale, Neighbors and Sobal [49] found that 68% of US male university students were dissatisfied with their current body shape as 20% wished to be heavier, and 48% preferred to be thinner. Mayo and George [39] observed a positive correlation between EAT-26 scores and body fat dissatisfaction as determined by the BIG among US college-aged men with a negative correlation between EAT-26 scores and muscle dissatisfaction.

Body dissatisfaction among college-aged Kuwaiti men may stem from several triggers. Previous work has shown that college years are a period when young adults can experience deterioration in self-concept, leading to anxiety, emotional distress, or depression [28, 65]. As college students transition from small high school communities to large universities, they could seek to change their outward appearance to an acceptable body shape that meets the local social requirements [15, 35]. The average age of marriage for Kuwaiti men and women is during their twenties [14], which matches the mean age of the participants. Early adulthood is associated with the decision to choose a suitable partner and paying special attention to appearance in comparison to others [66]. Some studies show that those seeking romantic relationships are more likely to develop disordered eating attitudes [24, 34]. Studies have reported high usage of AAS among male gym-goers, which could be linked to an increased desire to improve physical appearance [42]. In a study of 194 Kuwaiti male gym-goers aged from 14 to over 40 years old, 22.7% of the general sample and 4.2% of college students reported using AAS [7]. Most of the AAS users (70%) believed that an optimally muscular body can only be achieved by using AAS despite being well informed about harmful side effects [7]. The prevalence of overweight and obesity in the present study was 33 and 17.8%, respectively. This prevalence of obesity is much lower than the 54.2% prevalence reported by Musaiger et al. [45] in a sample of 203 Kuwaiti men. Likewise, Ng, Zaghloul, Ali, Harrison, and Popkin [51] found that the prevalence of obesity in a nationwide survey of adult men was 36% and overweight prevalence was 74%. Such high rates of obesity and overweight could contribute to the high degree of body dissatisfaction and disordered eating attitudes in the Kuwaiti male college students.

Our data showed that significantly greater proportions of students in arts-related majors were at risk of disordered eating attitudes than those in science-related majors. This finding is similar to a recent study in Palestine (2018), which found that academic specialization in faculties related to humanities was significantly associated with high EAT-26 scores [55]. The authors’ interpretation of this finding was that female humanities students tend to have more free time to watch television advertisements and use social media, as well as for activities focusing on beauty and body image than students in science- and engineering-based fields, who tend to experience higher academic pressure and less free time. Students in medicine, engineering, and science have usually obtained high grades in high school and have high academic and professional ambitions, which tend to limit their emphasis on body weight, and use of social media regarding issues pertaining to beauty and body image. Further investigations are needed to determine if the above aspects are applicable to students in Kuwait.

The overall study strengths include the relatively large sample of college-aged men across both public and private universities in Kuwait, the high response rate. Limitations include the lack of validation using a sample of Kuwaiti college students and lack of comparison with a clinically diagnosed sample with eating disorders for EAT-26 or muscle dysmorphia for BIG, respectively. Such limitations, however, are similar to most studies that have used both these instruments among college students. Validation studies are also required for both sexes. It is also important to understand how the psychometric properties of these measures extend cross-culturally in the Arabic context. Data on the measures of attractiveness (most attractive to the opposite sex) were beyond the scope of this paper, but these data may be useful in future studies evaluating whether the pressure to have a certain body type results from preference by potential sexual partners or from other sources such as media descriptions [32]. Another limitation is the reliance on the use of self-reporting for the main variables including BMI estimates; however, Spencer, Appleby, Davey, and Key [59] have reported a strong correlation between self-reported and objectively measured height and weight (*r* > .9). This high self-report reliability was confirmed in our pilot study [4], which showed highly significant correlations between actual weights and heights as measured by trained research assistants and self-reported weights and heights (Spearman’s rank *r* = .94 and .90, respectively).

## Conclusion

In summary, the present study highlights disordered eating attitudes and body dissatisfaction to be a significant problem among male college students in Kuwait. About half of the participants were at risk of disordered eating attitudes, and about three-quarters were dissatisfied with their body shape, regardless of their weight category. Such a high prevalence of disordered eating attitudes and behaviors emphasizes the need for research to identify the underlying risk factors to understand this emerging public health problem and so facilitate prevention activities on college campuses. Future studies of male college students need also to take into consideration assessment of purging behaviors, physical activity, dieting history, current dieting status, use of diet pills, and depression.

## Data Availability

Data are available from the corresponding author upon reasonable request.

## Abbreviations

AAS: Anabolic-Androgenic Steroids
BIG: Bodybuilder image grid
BMI: Body mass index
EAT-26: Eating attitudes test
FFMI: Fat free mass index
H: Height
M: Mean
ORs: Odds ratios
SD: Standard deviation
Sub-B: Subscale of Bulimia
Sub-D: Subscale of dieting
Sub-O: Subscale of oral control
W: Weight
